# Personalized Care in Eye Health: Exploring Opportunities, Challenges, and the Road Ahead for Chatbots

**DOI:** 10.3390/jpm13121679

**Published:** 2023-12-02

**Authors:** Mantapond Ittarat, Wisit Cheungpasitporn, Sunee Chansangpetch

**Affiliations:** 1 Surin Hospital and Surin Medical Education Center, Suranaree University of Technology, Surin 32000, Thailand; mantapond.sur@cpird.in.th; 2 Department of Medicine, Mayo Clinic, Rochester, MN 55905, USA; 3 Center of Excellence in Glaucoma, Chulalongkorn University, Bangkok 10330, Thailand; sunee.ch@chula.ac.th; 4 Department of Ophthalmology, Faculty of Medicine, Chulalongkorn University and King Chulalongkorn Memorial Hospital, Thai Red Cross Society, Bangkok 10330, Thailand

**Keywords:** ophthalmology, artificial intelligence, machine learning, language processing, large language models, chatbot, ChatGPT

## Abstract

In modern eye care, the adoption of ophthalmology chatbots stands out as a pivotal technological progression. These digital assistants present numerous benefits, such as better access to vital information, heightened patient interaction, and streamlined triaging. Recent evaluations have highlighted their performance in both the triage of ophthalmology conditions and ophthalmology knowledge assessment, underscoring their potential and areas for improvement. However, assimilating these chatbots into the prevailing healthcare infrastructures brings challenges. These encompass ethical dilemmas, legal compliance, seamless integration with electronic health records (EHR), and fostering effective dialogue with medical professionals. Addressing these challenges necessitates the creation of bespoke standards and protocols for ophthalmology chatbots. The horizon for these chatbots is illuminated by advancements and anticipated innovations, poised to redefine the delivery of eye care. The synergy of artificial intelligence (AI) and machine learning (ML) with chatbots amplifies their diagnostic prowess. Additionally, their capability to adapt linguistically and culturally ensures they can cater to a global patient demographic. In this article, we explore in detail the utilization of chatbots in ophthalmology, examining their accuracy, reliability, data protection, security, transparency, potential algorithmic biases, and ethical considerations. We provide a comprehensive review of their roles in the triage of ophthalmology conditions and knowledge assessment, emphasizing their significance and future potential in the field.

## 1. Introduction to the Utilization of Chatbots for Ophthalmology 

### 1.1. Overview of Chatbot Technology 

The rise of chatbot technology, as showcased by industry leaders like ChatGPT by OpenAI, Google’s Bard AI, Microsoft’s BingChat, and Anthropic’s Claude AI, has been a focal point of interest across myriad sectors, notably within the healthcare domain [1,2,3,4,5,6]. These chatbots are applications powered by artificial intelligence, meticulously designed to simulate human conversation through either text or voice interactions [4,7,8,9,10]. Utilizing cutting-edge natural language processing algorithms, these advanced systems can discern and address user inquiries with precision, delivering bespoke and pertinent information [1,2,11].

In the context of ophthalmology, chatbots introduce a fresh and innovative approach to delivering healthcare services, engaging with patients, and providing support to healthcare professionals [12,13,14]. Through harnessing the potential of chatbot technology, ophthalmology practices have the opportunity to augment accessibility, operational efficiency, and overall patient experience. For instance, integrating chatbots into their systems enables ophthalmology practices to deliver round-the-clock support, address common inquiries regarding eye health, facilitate appointment scheduling, and even offer preliminary guidance concerning eye conditions.

**Figure 1:**
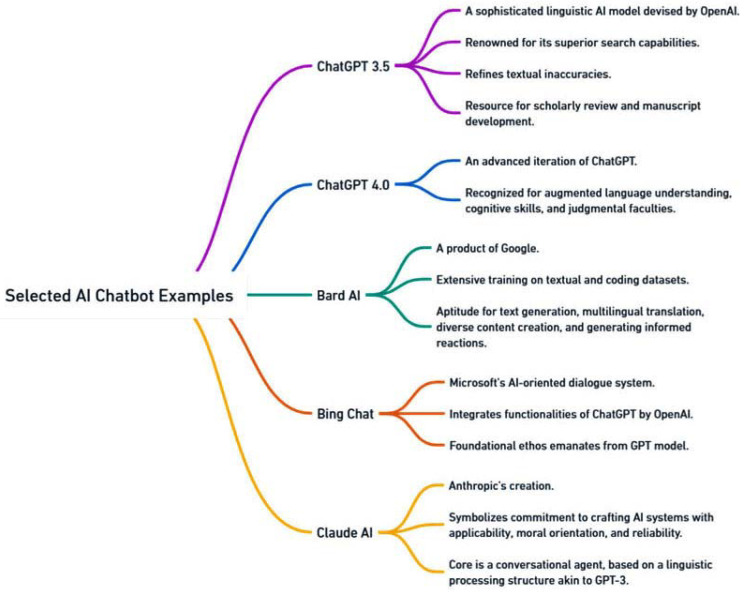
Examples of notable AI chatbots.

In the expansive landscape of ophthalmology, ChatGPT and chatbots of its ilk have heralded an era of uninterrupted communication, instantaneous information retrieval, and tailor-made interactions. These tools equip patients with the means to secure immediate help, while empowering healthcare practitioners to dispense proficient support. In streamlining the triage of patient queries, providing educational materials, and shepherding patients through both pre- and post-operative care directions, these chatbots have carved an indispensable niche for themselves. A clear demonstration of the influence of ChatGPT, which was developed by OpenAI, is found in the domain of medical pedagogy. Within this scope, it functions as an enhancer of search efficiency and a rectifier of manuscript inconsistencies. ChatGPT has emerged as an invaluable asset, especially in accessing specialized literature germane to renal transplant care. Equally impactful are other AI-driven conversational platforms, such as Microsoft’s BingChat and Google’s Bard AI. These technologically adept interfaces excel in enhancing search capabilities, remedying typographical and grammatical oversights, and enhancing the scrutiny of academic content [15]. Bard AI, with its rich foundational training in a myriad of texts and coding paradigms, is poised to craft context-sensitive textual interpretations [16]. This prowess positions it as an invaluable asset in healthcare, ranging from buttressing decisions anchored in evidence to honing the quality of communication (Figure 1).

The incorporation of chatbot technology in ophthalmology represents a promising advancement, capable of reshaping the provision of eye care by improving accessibility, operational efficiency, and patient satisfaction. Utilizing the capabilities of chatbots enables ophthalmology practices to provide a higher standard of care, improve patient outcomes, and furnish individuals with essential information and support.

The scoping review methodology used in our study has provided a detailed panorama of the current and potential applications of chatbot technology in ophthalmology, underscoring its role in enhancing patient engagement and improving care delivery. This comprehensive understanding is vital for formulating strategies that effectively incorporate chatbots into ophthalmological practices, thereby meeting the dynamic needs of patients and healthcare professionals in this field.

### 1.2. The Growing Need for Innovative Solutions in Ophthalmology

**Figure 2:**
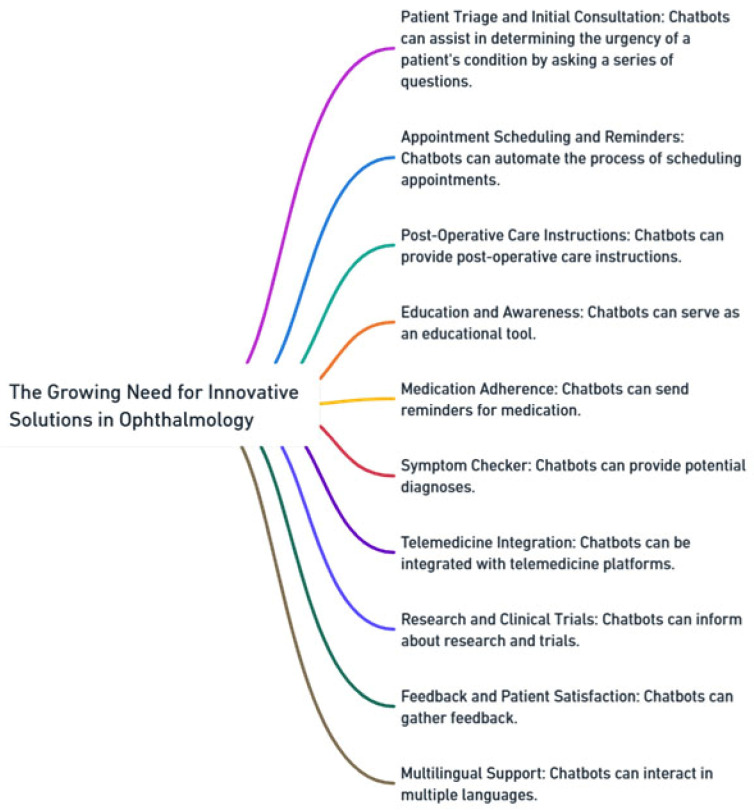
The growing need for innovative solutions in ophthalmology.

As the incidence of eye conditions increases, so does the demand for eye care services. This upswing highlights the urgent need for innovative solutions that can enhance healthcare delivery in the field of ophthalmology. The traditional methods, while effective, may not be sufficient to cater for the increasing number of patients requiring attention, especially in a timely manner. This is where technology, particularly chatbots, can make a significant difference [17,18], Figure 2.

Chatbots have the potential to play a pivotal role in ophthalmology by providing instantaneous access to information. One of their primary uses lies in patient triage and initial consultation. By asking a series of targeted questions, chatbots can assist in determining the urgency of a patient’s condition, thereby helping to prioritize those who need immediate attention. Additionally, these intelligent systems can automate the process of scheduling appointments, send timely reminders, and provide pre-appointment instructions. This ensures that patients are well-prepared for their visit, reducing wait times and enhancing the overall efficiency of the healthcare system.

Post-operative care is crucial in ophthalmology, especially after procedures like cataract surgery or LASIK. Chatbots can step in to provide patients with detailed post-operative care instructions, significantly reducing the risk of complications. Beyond this, they serve as an invaluable educational tool. Patients can receive information on common eye conditions, preventive measures, and general eye health tips directly from chatbots. This not only improves awareness but also empowers patients to take proactive steps in their healthcare journey. Furthermore, for chronic conditions where medication adherence is crucial, chatbots can send reminders, ensuring consistent medication use.

The integration of chatbots extends beyond basic patient interactions and doctor–patient consultations. They can be seamlessly integrated with various telemedicine platforms, facilitating a range of services such as medical video consultations for patient benefit, tele-reporting, administrative medical-health tele-consultancy, and tele-assistance for data transmission from ambulances to hospitals. This versatility enhances the efficiency and accessibility of healthcare services, making telemedicine more responsive to patient needs and healthcare dynamics. This is particularly beneficial in remote areas where immediate physical consultation might not be feasible. Additionally, chatbots can inform patients about ongoing research and clinical trials, aiding in participant recruitment and preliminary data gathering. They can also play a role in gathering feedback post-consultation or post-surgery, offering ophthalmologists insights into areas for improvement. Lastly, with multilingual support, chatbots ensure that language barriers do not impede the provision of quality care, making healthcare more inclusive and accessible.

## 2. Design and Development of Ophthalmology Chatbots 

### 2.1. Understanding User Needs and Requirements

**Figure 3:**
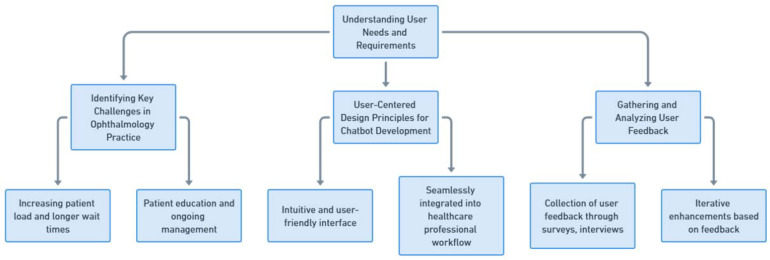
Understanding user needs and requirements.

When designing and developing chatbots for ophthalmology, it is critical to have an extensive understanding of the unique needs and requirements of users specific to the ophthalmology practice [19]. This includes considering the particular challenges faced in ophthalmology and adhering to user-centered design principles to ensure the creation of effective chatbots. The development process should also incorporate the collection and analysis of user feedback, to confirm that the chatbot meets the expectations and requirements of both patients and professionals in the discipline. Importantly, this entails creating two distinct paths in chatbot development: one tailored for doctors, using scientific and medical terminologies, and another for patients, employing non-medical, layperson-friendly language. This bifurcation ensures that the chatbot effectively communicates and engages with each group according to their specific knowledge levels and communication preferences (Figure 3).

#### 2.1.1. Identifying Key Challenges in Ophthalmology Practice

The practice of ophthalmology is confronted with a variety of unique challenges that can be effectively addressed through the integration of chatbot technology. One notable challenge lies in the increasing patient load stemming from the escalating prevalence of eye conditions, [17] resulting in longer wait times and limited availability of consultations [20,21,22]. Chatbots offer a potential solution to this challenge by providing automated symptom assessment and triage capabilities, enabling patients to promptly receive initial guidance concerning their conditions. However, it is crucial to underline that automatic triage systems have limitations and should not replace professional medical evaluation, especially in complex cases. For instance, distinguishing between initial herpetic keratitis, where cortisone is contraindicated, and conjunctivitis, where cortisone may be prescribed, requires precise symptom assessment and medical expertise that a chatbot may not reliably provide. Therefore, while chatbots can assist in preliminary guidance, they should be used in conjunction with, and not as a substitute for, professional medical advice.

Another significant challenge pertains to patient education. Many eye conditions necessitate ongoing management and patient compliance, which can be improved through the provision of effective education and information [23,24,25,26]. Ophthalmology chatbots have the ability to dispense general eye health information, elucidate common ophthalmic procedures and treatments, and offer guidance regarding pre- and post-operative care. This empowers patients to take an active role in their eye health, leading to improved adherence and better treatment outcomes.

#### 2.1.2. User-Centered Design Principles for Chatbot Development

The development of chatbots for ophthalmology necessitates adherence to user-centered design principles. This approach entails gaining a deep understanding of the specific needs, preferences, and behaviors of both patients and healthcare professionals operating within the field of ophthalmology.

Regarding patients, it is essential for the chatbot interface to be intuitive and user-friendly, featuring clear instructions and prompts, and avoiding complex medical terminology. Moreover, visual design elements should be optimized with ophthalmology in mind, taking into consideration factors such as color contrast, font size, and readability, to ensure inclusivity and accessibility for users with visual impairments [27].

As for healthcare professionals, the chatbot should be seamlessly integrated into their workflow. It should provide relevant and concise information, assist in the process of decision-making, and grant access to reference materials. Additionally, the chatbot should facilitate tasks such as appointment scheduling and reminders, streamlining the administrative responsibilities of healthcare professionals.

#### 2.1.3. Gathering and Analyzing User Feedback

The collection of user feedback plays a critical role in refining and enhancing the design and functionality of ophthalmology chatbots. User feedback can be obtained through various channels, such as surveys, interviews, and user testing sessions. This valuable input provides insights into UX, challenges encountered, and areas in need of improvement.

The analysis of user feedback enables iterative enhancements of the chatbot’s performance. It aids in the identification of common issues, understanding user preferences, and more importantly, uncovering any gaps in the chatbot’s capabilities. This is achieved by systematically analyzing feedback for patterns of misunderstandings, incorrect responses, or inadequate information provided by the chatbot. For instance, if multiple users report confusion over a particular set of symptoms or express dissatisfaction with the guidance provided, this indicates a gap in the chatbot’s knowledge or in its ability to interpret user inputs accurately. Additionally, feedback can highlight areas where the chatbot’s communication style is not effective or user-friendly. This iterative process ensures that the chatbot aligns with user needs, ultimately enhancing its effectiveness and overall user satisfaction.

### 2.2. Chatbot Architecture and Functionality 

**Figure 4:**
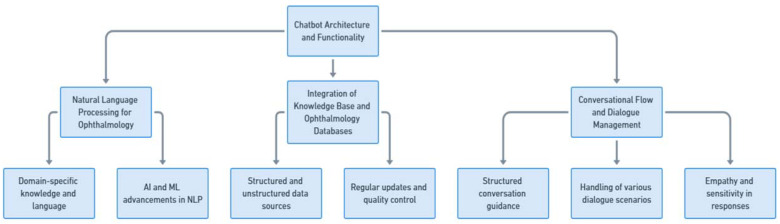
Chatbot architecture and functionality.

In the context of ophthalmology chatbots, it is of importance to have a well-designed and functional architecture that specifically caters to the unique requirements of the field. This section thoroughly examines three pivotal aspects: natural language processing (NLP) tailored for ophthalmology, the integration of the knowledge base and medical databases, and conversational flow and dialogue management (Figure 4).

#### 2.2.1. Natural Language Processing for Ophthalmology 

Within the ophthalmology domain, NLP technology must be tailored to the specific language and terminology used. Accurate recognition and comprehension of ophthalmic terms, medical abbreviations, and anatomical references by the chatbot are essential. Moreover, NLP algorithms must be equipped to handle the complexity of ophthalmic queries, which often involve specific symptoms, laterality, and ophthalmic investigations. The chatbot should possess the capability to extract pertinent information from user inputs and generate appropriate responses. Additionally, it should demonstrate an understanding of the context of the conversation, thereby facilitating more meaningful interactions.

To attain these capabilities, the development of ophthalmology chatbots necessitates an in-depth understanding of domain-specific knowledge and language. Incorporating domain-specific ontologies, medical literature, and expert knowledge can significantly enhance the accuracy and effectiveness of the employed NLP algorithms. With the development of AI and ML, NLP in ophthalmology has evolved significantly in recent years, encompassing text data extraction, part-of-speech tagging, indexing, tokenization, classification, entity recognition, and word embeddings [28]. This has enabled Chatbot development to achieve desirable features.

#### 2.2.2. Integration of Knowledge Base and Ophthalmology Databases 

The integration of the knowledge base and ophthalmology databases is pivotal for ophthalmology chatbots to provide accurate and up-to-date information. It is imperative that this information be readily accessible to the chatbot, facilitating the delivery of reliable responses and recommendations.

The knowledge base can encompass structured information such as clinical guidelines, best practices, and standardized treatment protocols. These protocols are typically derived from clinical trials and the consensus among medical experts. However, it is important to acknowledge that clinical trials can sometimes yield conflicting results. In such cases, the role of comprehensive databases like PubMed becomes crucial. PubMed serves as a repository of diverse medical literature, allowing chatbots to access a wide range of research articles, case studies, and meta-analyses. This enables the chatbot to incorporate the most current and widely accepted medical knowledge, while also considering differing viewpoints and emerging research. Additionally, the integration of medical databases enables the chatbot to access patient-specific data, empowering it to provide personalized recommendations based on individual patient characteristics and specific eye conditions.

To ensure the accuracy and reliability of the information, regular updates and quality control measures should be implemented. Collaboration with ophthalmology experts, clinicians, and researchers is essential to validate and maintain the data sources.

#### 2.2.3. Conversational Flow and Dialogue Management

An effective ophthalmology chatbot should possess the ability to manage conversational flow and dialogue in a seamless and natural manner. The dialogue management system orchestrates the interaction between the chatbot and the user, ensuring smooth transitions and relevant responses.

A chatbot is designed to engage in comprehensive conversations with patients, effectively addressing a wide range of concerns and queries. This includes guiding users through structured dialogues to gather necessary medical information; handling various dialogue scenarios, such as clarifying ambiguous queries; asking pertinent follow-up questions; and providing clear, detailed explanations. Importantly, the chatbot should be equipped to discuss treatment options and medical advice, tailoring its responses to the individual’s medical history and current health status.

In addition to general inquiries, the chatbot must be adept at managing interruptions, context switches, and multi-turn conversations, thereby enabling a more natural and user-friendly interaction. It should exhibit empathy and sensitivity in its responses, considering the emotional aspects of patients’ discussions, which is crucial in conversations about treatment and health concerns. The utilization of advanced language generation techniques aids in creating responses that are not only informative and compassionate, but also easily comprehensible to patients from diverse backgrounds. Continuous testing and analysis of user feedback are essential for optimizing conversational flow and dialogue management, ensuring that the chatbot remains effective in both general and treatment-specific discussions.

### 2.3. Chatbot User Interface and User Experience Design

**Figure 5:**
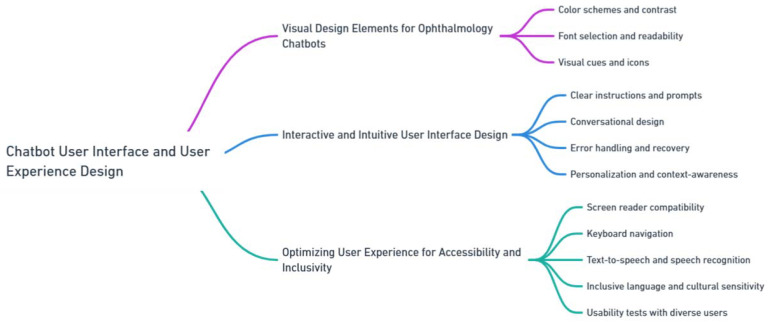
Chatbot user interface.

The creation of an effective user interface (UI) and the provision of a positive user experience (UX) are imperative for achieving optimal engagement and usability. This section emphasizes three fundamental aspects: visual design elements tailored specifically for ophthalmology chatbots, interactive and intuitive UI design, and the optimization of UX with regard to accessibility and inclusivity (Figure 5).

#### 2.3.1. Visual Design Elements for Ophthalmology Chatbots

Visual design elements play a pivotal role in the development of an engaging and user-friendly UI for ophthalmology chatbots. To ensure these elements are effectively customized, it is imperative that the chatbot first gathers and understands the distinctive characteristics and requirements of each patient in the ophthalmology practice.

Color schemes and contrast are key aspects of this customization. Selecting appropriate color schemes and ensuring suitable contrast levels are vital for enhancing readability and visual comfort, especially for users with visual impairments. The chatbot must be capable of adapting its interface based on the specific visual needs of the patient. For example, utilizing high-contrast colors for text and background elements can significantly improve readability for individuals with low vision. This adaptive approach ensures that the chatbot’s UI is not only visually appealing but also tailored to meet the unique needs of each patient, thereby providing a more personalized and effective user experience [27].

Font selection and readability: Employing clear and easily readable fonts facilitates effortless navigation for users of the chatbot interface. Additionally, incorporating font sizes that can be easily adjusted enables users to customize the display according to their specific needs.

Visual cues and icons: The integration of visual cues and icons contributes to an enhanced UX overall. Utilizing intuitive icons and symbols that are specific to ophthalmology, such as eye-related illustrations or medical symbols, assists in quickly conveying information and guiding users through the chatbot interface.

#### 2.3.2. Interactive and Intuitive User Interface Design

Creating an interactive and intuitive UI is pivotal for ophthalmology chatbots to effectively engage and assist users. The UI design should enable seamless navigation and provide a user-friendly experience [29]. 

Clear instructions and prompts: Chatbots should employ explicit instructions and prompts to guide users throughout their interactions. Offering step-by-step guidance and clear instructions on how to interact with the chatbot facilitates a smooth flow of conversation.

Conversational design: Emulating natural conversation in a chatbot interface is crucial for enhancing user engagement. The chatbot should provide a conversational tone, mimicking human-like interactions, and appropriate responses to user inputs, including variations in language, phrasing, and sentence structure.

Error handling and recovery: To maintain a positive user UX, the chatbot should adeptly handle user errors or misunderstandings, provide suggestions for correcting or rephrasing queries, and offer help options for users to recover from errors or confusion.

Personalization and context-awareness: Personalization and context-awareness in UI design can enhance the UX by allowing chatbot to remember user preferences, past interactions, and relevant information. This facilitates a more personalized and tailored conversation, providing users with a sense of continuity and familiarity.

#### 2.3.3. Optimizing User Experience for Accessibility and Inclusivity

Ensuring accessibility and inclusivity in the design of ophthalmology chatbots is of importance. The UI must be designed to accommodate the diverse needs of users, including those with visual impairments or disabilities.

Ensuring the compatibility of the chatbot interface with screen readers and other assistive technologies is a critical aspect of our design, particularly for users with visual impairments. To achieve this, a chatbot should be regularly evaluated and updated by a dedicated accessibility team, which includes experts in assistive technology, user experience designers, and representatives from the visually impaired community. This team is responsible for providing text alternatives for visual elements and incorporating appropriate semantic markup, enabling screen readers to effectively interpret and convey information.

Another key feature of chatbot accessibility is enabling keyboard navigation, which is essential for users who rely solely on keyboard interactions. The chatbot interface should be designed to allow users to navigate through the conversation and access all functionalities using keyboard commands. Modifications to keyboard navigation are also overseen by an accessibility team, which bases changes on user feedback, technological advancements, and best practices in digital accessibility.

Text-to-speech and speech recognition: Integration of text-to-speech and speech recognition capabilities enhances accessibility for users with visual or motor impairments. This feature enables users to interact with the chatbot through voice commands and receive audio responses.

Inclusive language and cultural sensitivity: The language used by the chatbot should be inclusive and culturally sensitive. It should avoid biased language and be designed to cater to users from diverse cultural and linguistic backgrounds. The utilization of natural language generation techniques assists in generating inclusive and respectful responses.

To optimize UX for accessibility and inclusivity, it is crucial to conduct usability tests with a diverse group of users, including individuals with disabilities. Gathering feedback and incorporating suggested improvements ensures that the chatbot interface meets the needs of a wide range of users.

By adhering to these design principles and focusing on visual elements, interactive UI, and accessibility, ophthalmology chatbots can provide a seamless and inclusive UX.

## 3. Applications and Benefits of Chatbots in Ophthalmology

### 3.1. Remote Patient Monitoring and Triage

**Figure 6:**
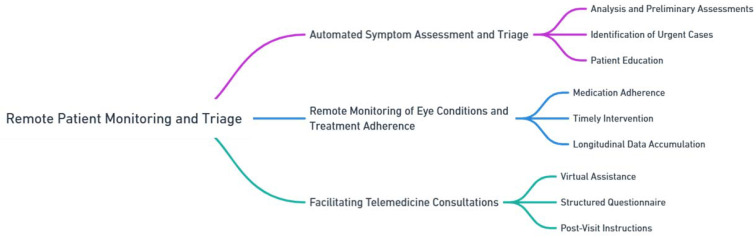
Remote patient monitoring and triage in ophthalmology.

In the field of ophthalmology, chatbots have emerged as valuable tools for remote patient monitoring and triage, providing numerous advantages for both patients and healthcare providers. This section explores three primary applications of chatbots in ophthalmology, namely automated symptom assessment and triage, remote monitoring of eye conditions and treatment adherence, and facilitating telemedicine consultations (Figure 6).

#### 3.1.1. Automated Symptom Assessment and Triage

Chatbots equipped with advanced NLP capabilities can assess and triage ophthalmic symptoms. Patients can interact with the chatbot, describing their symptoms and providing relevant information. The chatbot then analyzes this input and generates preliminary assessments based on established medical guidelines and protocols.

An ophthalmology chatbot is designed to perform automated symptom assessment and triage, enabling the timely identification of urgent cases, such as acute vision loss or severe eye pain. This system utilizes a sophisticated algorithm, developed in collaboration with ophthalmology experts, which assesses the severity and nature of symptoms reported by the patient. Urgent cases are identified based on predefined criteria, such as the sudden onset of symptoms, intensity of pain, or risk factors for serious eye conditions.

For non-urgent cases, the chatbot provides appropriate care recommendations. These may include self-care advice for minor symptoms or guidance to schedule a routine appointment with an ophthalmologist. These recommendations are based on established clinical guidelines and tailored to the individual’s reported symptoms. This prioritization not only helps in preventing irreversible vision impairment or complications in urgent cases but also ensures that patients with less severe symptoms receive the most suitable care advice. This approach optimizes healthcare resources and reduces unnecessary visits to ophthalmology clinics or emergency departments.

Furthermore, the automated symptom assessment and triage conducted through chatbots contribute to patient education. The chatbot can provide information about common ophthalmic conditions, preventive measures, and self-management strategies. This empowers patients to make informed decisions about their eye health and promotes active participation in their own care.

#### 3.1.2. Remote Monitoring of Eye Conditions and Treatment Adherence

Ophthalmology chatbots can assist in the remote monitoring of eye conditions and ensuring treatment adherence in patients with chronic eye conditions. Through regular interactions with patients, chatbots can gather information about visual symptoms, medication usage, and lifestyle factors that may impact eye health. Chatbots can improve medication adherence for patients with complex eye medication regimens. By sending reminders, educational messages, and addressing common concerns, chatbots can improve understanding and compliance, ensuring better treatment outcomes.

The remote monitoring capabilities of ophthalmology chatbots are crucial for timely intervention in cases of disease progression or non-adherence to treatment. In this context, “high-risk situations” refer to scenarios where there is a significant risk of rapid disease progression, potential vision loss, or other serious complications. These situations may include, but are not limited to, sudden changes in vision, symptoms indicating potential retinal detachment, or signs of acute glaucoma.

Healthcare providers, including ophthalmologists, can be alerted by the chatbot to these high-risk situations, allowing for proactive management and prevention of complications. This feature is particularly beneficial for patients in remote areas or those with limited access to healthcare, as it reduces the need for travel and associated costs. Furthermore, remote monitoring facilitates the accumulation of longitudinal data on patients’ eye health, which can be invaluable for research purposes, population health management, and the improvement of treatment protocols.

#### 3.1.3. Facilitating Telemedicine Consultations

The integration of chatbots in ophthalmology facilitates telemedicine consultations, enabling remote access to specialized eye care. While telemedicine has gained prominence, especially in situations where physical visits are challenging, it is important to recognize the current limitations of chatbots in this context. Chatbots may serve as virtual assistants during telemedicine consultations, providing support to ophthalmologists and enhancing the patient experience. Patients can engage with the chatbot for tasks like providing medical history and addressing preliminary concerns. However, we acknowledge that in the current implementation, chatbots do not support the sharing of images, which is a significant aspect of telemedicine in ophthalmology. For instance, a patient describing symptoms of a red eye could provide much more diagnostic value through an image, which a chatbot currently cannot process.

Prior to the telemedicine consultation, the chatbot can guide patients through a structured questionnaire to collect essential information. However, the integration of image-sharing capabilities in future iterations could greatly enhance the diagnostic process. After the consultation, the chatbot provides post-visit instructions and resources, but the addition of image analysis could further personalize and improve post-visit care. We recognize the importance of image exchange in ophthalmology and anticipate future advancements in chatbot technology that will enable this functionality, thereby significantly enhancing the effectiveness of telemedicine consultations in this field.

### 3.2. Patient Education and Information Provision

Within the field of ophthalmology, chatbots have emerged as effective tools for providing patient education and information (Table 1). This section examines three key aspects of patient education and information provision facilitated by ophthalmology chatbots, namely the dispensing of general eye health information, the explanation of common ophthalmic procedures and treatments, and the provision of guidance on pre- and post-operative care.

#### 3.2.1. Dispensing General Eye Health Information

Ophthalmology chatbots can serve as interactive platforms that grant patients access to accurate and up-to-date information [30]. Through engagement with the chatbot, patients can obtain tailored information based on their individual needs and concerns. Moreover, it can educate patients on the significance of regular eye examinations and lifestyle factors such as protection from ultraviolet (UV) radiation.

**Figure 7:**
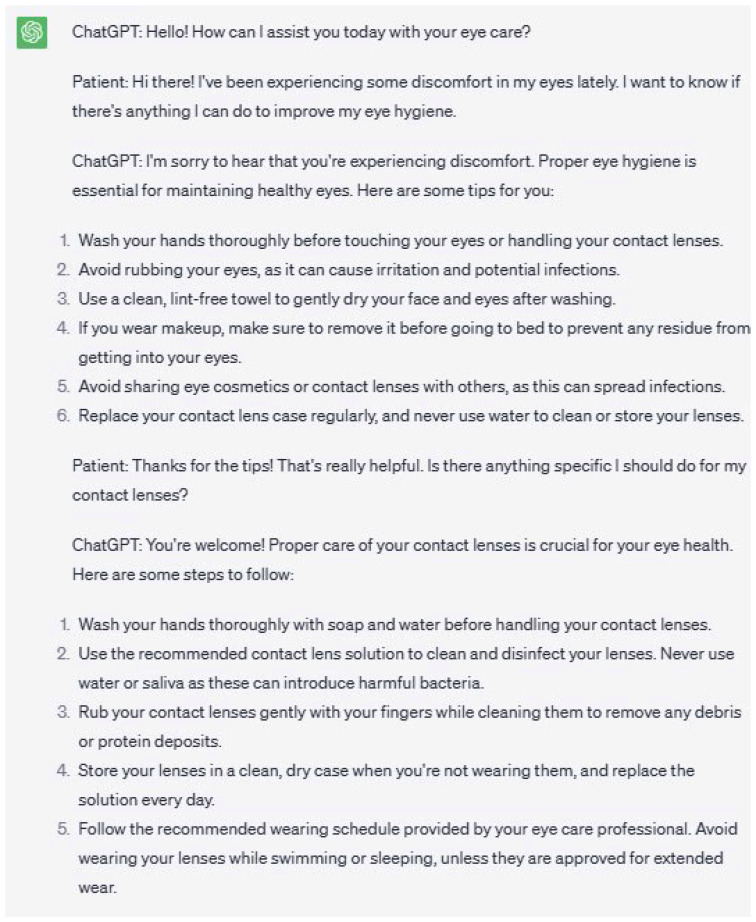
This figure illustrates a two-way conversation between a patient and ChatGPT, a language model designed to offer guidance on eye care topics. The patient seeks advice regarding discomfort in their eyes and ways to improve eye hygiene. ChatGPT responds by providing concise and comprehensible information, including tips on proper eye hygiene and steps for the care of contact lenses. The chatbot adapts its responses to accommodate the patient’s level of understanding, making the conversation informative and helpful for the patient’s eye health.

Chatbots can address frequently asked questions, debunk myths and misconceptions, and clarify doubts related to eye health. They can offer guidance on topics such as proper eye hygiene, contact lens care, and vision correction options (Figure 7). The chatbot is capable of adapting its responses to accommodate the patient’s level of understanding and of delivering information in a concise and comprehensible manner.

Dispensing general eye health information through chatbots fosters patient engagement and active participation in eye care. They encourage proactive measures and reduce reliance on traditional sources such as pamphlets or websites. Patients can receive personalized information and engage in interactive conversations with the chatbot, thereby enhancing their overall learning experience.

#### 3.2.2. Explanation of Common Ophthalmic Procedures and Treatments

Ophthalmology chatbots serve as valuable tools for elucidating common ophthalmic procedures and treatments to patients. They provide detailed information about diagnostic tests, surgical interventions, and medical treatments in a user-friendly and accessible manner.

Patients can interact with the chatbot to acquire knowledge about the purpose, process, and potential outcomes of various ophthalmic procedures. The chatbot can guide them through the steps involved in diagnostic tests such as visual acuity assessments, tonometry, and fundoscopy. Moreover, it can guide them in understanding surgical procedures like cataract surgery, LASIK, or corneal transplant, including the associated benefits, risks, and recovery process.

Chatbots help bridge the communication gap between patients and healthcare provider, allowing patients to review and reinforce their understanding of procedures and treatments at their own pace. They can educate patients about different treatment modalities for specific eye conditions. They can also elucidate the mechanisms of action and potential side effects of medications used in ophthalmology. Additionally, the explanation of common ophthalmic procedures and treatments enhances patient comprehension and reduces anxiety, by offering clear and accurate information. Patients gain a better understanding of the procedures or treatments they may undergo, thereby promoting informed decision-making and alleviating fears or uncertainties.

#### 3.2.3. Guidance on Pre- and Post-Operative Care

Adequate preparation of patients for the surgical experience and the provision of appropriate post-operative care are essential for optimizing outcomes and minimizing complications.

Prior to surgery, chatbots can recommend to patients the necessary preparations, which may include fasting requirements and medication adjustments. They can provide advice on what to expect during the procedure, address common concerns, and offer reassurance and support. It is important to note that while chatbots can provide these recommendations based on standard pre-surgical protocols, the final preparation plan may be modified by the surgeon as necessary, tailored to the individual patient’s condition. This ensures that patients receive personalized care, while also benefiting from the convenience and support offered by the chatbot.

Following the surgery, chatbots can offer comprehensive guidance on post-operative care, medication regimens, proper wound care, and the use of protective measures such as eye shields or patches. They can educate patients about common post-operative symptoms, signs of complications, and the need for follow-up appointments.

Gathering guidance on pre- and post-operative care through chatbots ensures that patients are well-informed, leading to improved compliance and better surgical outcomes. Clear instructions and guidance make patients more likely to adhere to the recommended care plans, thereby reducing the risk of complications and promoting successful recoveries. Additionally, chatbots can offer ongoing support and accessibility to patients during the post-operative period, reducing the need for unnecessary emergency visits and fostering continuity of care.

### 3.3. Support for Ophthalmology Professionals

**Figure 8:**
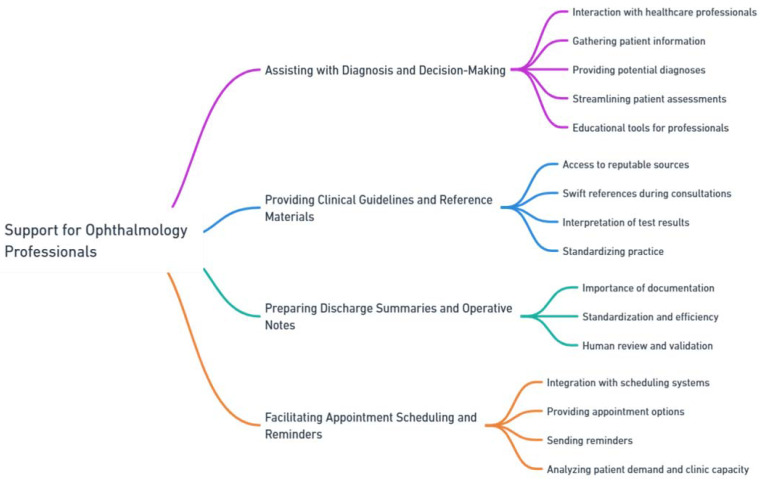
Support for ophthalmology professionals.

Ophthalmology chatbots provide assistance in diagnosis and decision-making, delivery of clinical guidelines and reference materials, as well as in managing appointment scheduling and reminders. This section offers a detailed examination of these key areas, emphasizing the benefits and enhancements they bring to healthcare professionals in ophthalmology (Figure 8). 

#### 3.3.1. Assisting with Diagnosis and Decision-Making

Ophthalmology chatbots can assume a pivotal role in assisting healthcare professionals with diagnosis and decision-making processes. By utilizing their conversational and data processing capabilities, these chatbots interact with healthcare professionals, assisting in the collection of pertinent information related to patients’ ocular conditions.

Chatbots can be programmed to pose targeted questions regarding patients’ symptoms, medical history, ocular examinations, and other factors that may relate to certain diseases. By assimilating this information, chatbots assist healthcare professionals in developing a comprehensive understanding of the patient’s condition, which can facilitate accurate diagnoses and informed treatment decisions. Additionally, chatbots can analyze the collected data and furnish healthcare professionals with potential diagnoses or differential diagnoses based on established clinical guidelines and algorithms, as well as integrating preexisting risk calculation tools, such as the age-related macular degeneration (AMD) risk calculator and the ocular hypertension treatment study (OHTS) risk calculator [31,32,33,34,35].

This serves as a valuable reference point for healthcare professionals, enabling them to make well-grounded and timely decisions. Ophthalmology chatbots can save healthcare professionals time by streamlining patient assessments and gathering relevant information. They also serve as valuable educational tools, providing up-to-date information on research findings, treatment options, and emerging trends, contributing to professional growth and enhancing expertise in the field.

#### 3.3.2. Providing Clinical Guidelines and Reference Materials

Ophthalmology chatbots possess the capability to furnish healthcare professionals with clinical guidelines and reference materials. These chatbots can be programmed to access and retrieve information from reputable sources, such as medical databases, clinical practice guidelines, and research articles.

By having access to an extensive array of information, healthcare professionals can employ chatbots as swift references during patient consultations. Chatbots provide evidence-based recommendations for the diagnosis, treatment, and management of various ocular conditions. They also offer guidelines for monitoring and follow-up care, ensuring that healthcare professionals remain abreast of best practices in ophthalmology. Moreover, chatbots assist healthcare professionals in interpreting test results and imaging studies. They provide explanations for various ophthalmic tests, such as visual field testing, optical coherence tomography (OCT), or fundus photography. This aids healthcare professionals in accurately interpreting results and making well-informed decisions regarding patient care.

The provision of clinical guidelines and reference materials by ophthalmology chatbots confers numerous benefits. First, it ensures that healthcare professionals have immediate access to reliable and evidence-based information. This supports the decision-making process and facilitates the delivery of high-quality care to patients.

Second, chatbots aid in standardizing practice and fostering consistency in care delivery. By providing guidelines and recommendations, chatbots assist healthcare professionals in adhering to established protocols and best practices. This fosters improved patient outcomes and enhances the quality of care across various healthcare settings.

#### 3.3.3. Preparing Discharge Summaries and Operative Notes

Discharge summaries and operative notes are of importance in ophthalmology for maintaining continuity of care, facilitating effective communication among healthcare providers, serving as legal documentation, supporting research and education, and promoting patient safety and quality improvement. While the significance of these factors is recognized, variations in content and the time-consuming process pose the greatest challenges in achieving excellence [36,37,38].

Using a chatbot for writing discharge summaries and operative notes can offer several advantages in terms of standardization, efficiency, accuracy, and convenience [39]. With proper training and the improvement of AI libraries, chatbots can be seen as tools to assist ophthalmology healthcare in generating comprehensive and efficient discharge summary and operative notes. It is important to note that human review and validation are crucial to ensure accuracy, especially in complex cases and for handling situations that require clinical judgement and empathy.

#### 3.3.4. Facilitating Appointment Scheduling and Reminders

Ophthalmology chatbots possess the capability to facilitate appointment scheduling and reminders for healthcare professionals. They can seamlessly integrate with existing scheduling systems and electronic health records, enabling patients to conveniently book appointments and receive timely reminders about their upcoming visits.

Chatbots provide patients with options for available appointment slots, assisting them in finding suitable times that align with their schedules. They also automatically send reminders to patients, reducing the likelihood of missed appointments and enhancing overall clinic efficiency. Patients can access the chatbot at their convenience, obviating the need for phone calls or waiting on hold to schedule appointments, thus enhancing patient satisfaction and engagement.

Furthermore, chatbots can aid healthcare professionals in managing their schedules by optimizing appointment bookings. Through analyzing patient demand and clinic capacity, chatbots suggest optimal scheduling strategies that minimize wait times and maximize clinic utilization. This enables healthcare professionals to streamline their workflow, for reduced administrative burden and increased productivity, to provide timely care to their patients.

### 3.4. Ophthalmology Training

Chatbots hold immense potential as valuable educational tools in medical training, offering accessible and interactive resources to learners [40]. In the context of ophthalmology training, chatbots can play a crucial role in various aspects, including providing the fundamentals of ophthalmology, facilitating case studies and diagnostic support, offering adaptive assessment, and even simulating surgical procedures. Furthermore, they can assist with administrative tasks and personalized course organization, tailoring the learning experience to individual needs. One of the most significant advantages of chatbots in this domain is their ability to provide comprehensive knowledge and reference materials essential for ophthalmology training. By harnessing AI’s continuous updating capabilities, these chatbots can ensure that learners access the most up-to-date information, thereby enhancing the overall learning experience.

Through incorporating simulated patient interactions, chatbots enable learners to practice and refine their clinical skills effectively. By presenting realistic case scenarios, students can engage in diagnostic decision-making and treatment proposals. The chatbot can then offer valuable feedback on their decisions, guiding them throughout the process. This feedback mechanism not only helps learners identify areas for improvement but also provides specific recommendations for additional study or practice, which can be invaluable for their professional growth. However, it is essential to acknowledge that there are still areas for improvement in the utilization of chatbots in ophthalmology training. One such aspect is the need to focus on enhancing accuracy. Ensuring that the chatbot’s responses are consistently reliable and aligned with established medical knowledge is crucial for the success of such educational tools.

## 4. Availability and Performance of Current Ophthalmology Chatbots

Chatbots have emerged as innovative tools in the field of ophthalmology. Chatbots, such as ChatGPT, can be integrated into various platforms such as web-based platforms, mobile applications, messaging applications, and virtual reality platforms. The following sections present examples of chatbot use in ophthalmology and summarize the current performance of chatbots employed in various ophthalmology-related contexts.

### 4.1. Chatbot Performance in Triage Ophthalmology Conditions

In a study performed by Tsui, J.C. et al., ten prompts reflecting common patient complaints related to common ophthalmology conditions were used to determine the suitability of ChatGPT 3.0 responses. The study also evaluated the precision of the responses by comparing the responses to the same questions from three individual chats. The study found a majority of responses were precise and suitable; however, 20% of responses were considered imprecise or unsuitable [41].

**Figure 9:**
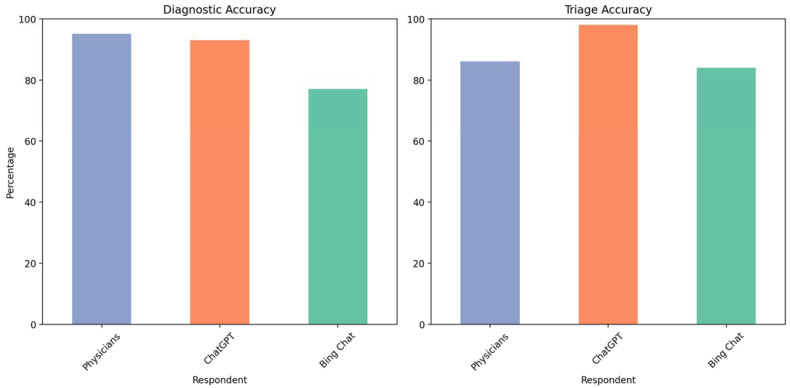
Chatbot performance in triage ophthalmology conditions. On the left, “correct diagnosis accuracy” bar chart; and on the right, “correct triage accuracy” bar chart.

A more recent study by Lyons, R.J. and associates also evaluated the triage performance of 44 vignettes representing common emergency room ophthalmologic diagnosis using three publicly available AI chatbots, namely ChatGPT 4.0, Bing Chat (Microsoft Corporation, Redmond, WA, USA), and WebMD Symptom Checker (WebMD Inc., New York, NY, USA). The responses from the chatbots were compared with physician respondents. The study found that ChatGPT using GPT-4 model yielded the highest diagnostic (93%) and triage (98%) accuracy (Figure 9). Although Bing resulted in a high accuracy of diagnosis, there were incorrect responses in 14% of cases, whereas none were discovered for ChatGPT [42]. 

### 4.2. ChatGPT Performance in Patient Education and Information Provision

A study from Potapenko et al. assessed the accuracy of patient information for five common retinal diseases (i.e., age-related macular degeneration, diabetic retinopathy, retinal vein occlusion, retinal artery occlusion, and central serous chorioretinopathy) using ChatGPT 3.0. They evaluated accuracies in disease summary, prevention, treatment options, and prognosis. Most responses showed high accuracy, with median ratings ranging from “good/only minor non-harmful inaccuracies” to “very good/no inaccuracies.” However, treatment options had “moderate/potentially misinterpretable inaccuracies”, with 12 of 100 treatment responses showing “potentially harmful inaccuracies” [7].

Similarly, another study focusing on patient information from ChatGPT 3.0 on vernal keratoconjunctivitis (VKC) also found that current ChatGPT responses, while relevant to typical questions, are still imperfect. The responses related to treatment and prevention received considerably lower accuracy ratings from the experts than VKC general, prognosis, and allergy-related questions. Inaccuracies such as missing essential information regarding potentially serious steroid side effects and harmful suggestions such as removal of conjunctiva were discovered [30].

### 4.3. Chatbots Examples in Supporting for Healthcare Professionals

A preliminary work utilizing ChatGPT 3.0 to generate discharge summaries across subspecialties found that the AI-constructed documents were able to shorten the processes; however, their quality was based on the completeness of the prompts given and required training and adjustment [39]. Table 2 shows an example of a discharge summary generated by ChatGPT.

**Figure 10:**
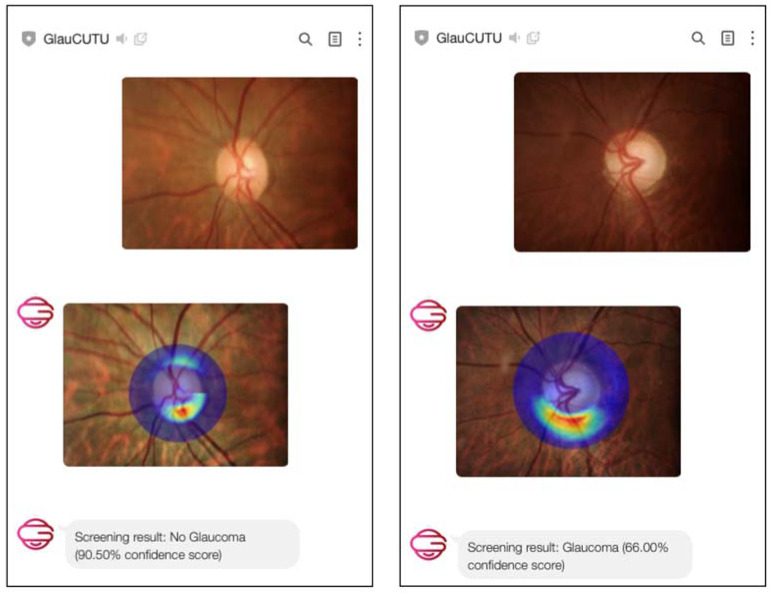
Example of responses generated by GlauCUTU illustrating glaucoma risk assessment from an optic disc photo.

GlauCUTU is another example of a chatbot designed to aid with glaucoma diagnosis. This chatbot utilizes a deep learning algorithm to provides real-time response to help in screening glaucoma based on optic disc photo [43]. GlauCUTU operates on the mobile and desktop social messaging service LINE (Figure 10). With the integration of a messaging application, it provides a convenient and readily accessible mode of communication and can be considered an example of an ophthalmologist virtual assistant [43].

### 4.4. Chatbot Performance in Ophthalmology Knowledge Assessment

**Figure 11:**
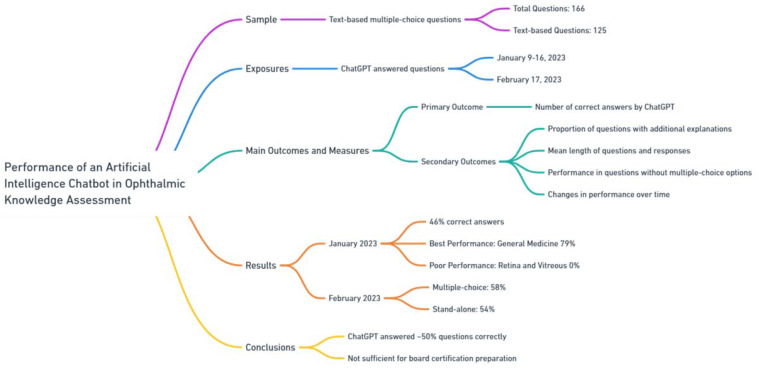
Performance of an artificial intelligence chatbot in ophthalmic knowledge assessment.

The performance of chatbots can vary across disciplines and different subspecialties (Table 3). While ChatGPT answered a majority of general medicine licensing examination questions correctly [44], the present version of ChatGPT did not correctly answer multiple-choice questions (MCQ) for the US board certification preparation (i.e., Ophthalmic Knowledge Assessment Program (OKAP) and Written Qualifying Exam (WQE) from the OphthoQuestions) to a desirable level. A study indicated that ChatGPT 3.0 correctly answered only 46% of 125 multiple-choice questions intended to prepare for board certification examinations [45] Figure 11. 

**Figure 12:**
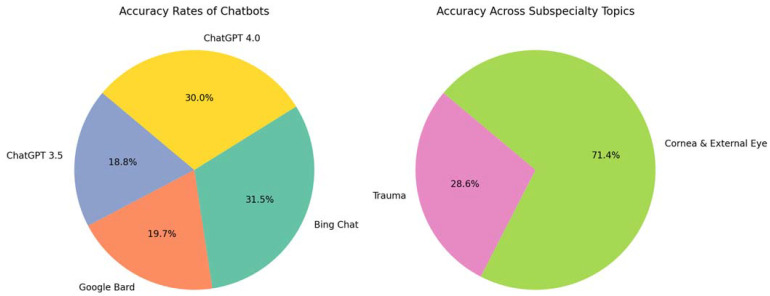
Comparative analysis of large language models in the Royal College of Ophthalmologists fellowship exams.

Another study compared more update versions of ChatGPT (ChatGPT 3.5 and 4.0) with Bing Chat and Google Bard (Alphabet Inc., CA, US) in their accuracy in answering the UK’s postgraduate MCQ exam for the Fellowship of Royal College of Ophthalmologists (FRCOphth) [46]. The study found accuracy rates of 49.6%, 51.9%, 82.9%, and 79.1% for ChatGPT 3.5, Google Bard, Bing Chat, and ChatGPT 4.0, respectively. However, the accuracy of ChatGPT 4.0 increased to 88.4% with prompting or tuning strategies. It should be noted that the accuracy varied widely across subspecialty topics with the lowest for trauma (accuracy 38.5%) and the highest for cornea and external eye (accuracy 96.2%), Figure 12.

**Figure 13:**
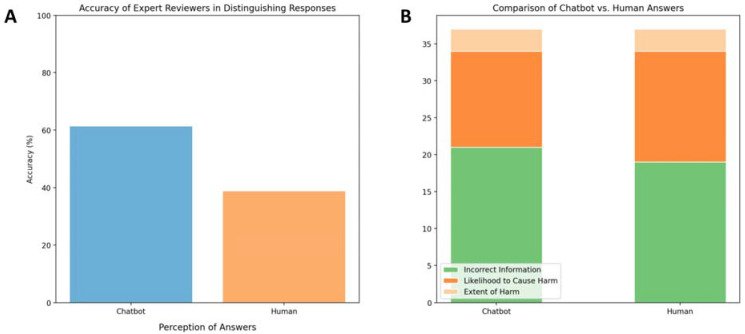
(**A**) Accuracy of expert reviewers in distinguishing responses. (**B**) Comparison of chatbot vs. human answers.

Recently, Bernstein et al. [47] conducted a cross-sectional study evaluating the quality of ophthalmology advice generated by ChatGPT, a large language model (LLM) chatbot, compared to advice written by ophthalmologists (Figure 13). The authors analyzed 200 pairs of online forum posts with patient eye care questions and responses by American Academy of Ophthalmology physicians. A panel of eight masked ophthalmologists were asked to distinguish between chatbot and human answers. The expert reviewers correctly identified chatbot vs. human responses with 61% accuracy on average (Figure 13A). However, the ratings of chatbot and human answers were comparable regarding the inclusion of incorrect information (21% vs. 19%), likelihood to cause harm (13% vs. 15%), and extent of harm (3% vs. 3%), (Figure 13B). The quality of chatbot answers was not rated as significantly inferior to human answers. The results suggest large language models may be capable of providing appropriate responses to patient eye care questions across a range of complexity. Further research is needed to evaluate the performance, ethics, and optimal clinical integration of chatbots in ophthalmology.

In ophthalmology, chatbots have the potential to be useful tools. The currently available chatbots are restricted in their availability and performance. Generally, they are able to provide acceptably broad knowledge and initial guidance. However, users must be mindful of their limitations, particularly in complicated settings where the proportion of incorrect chatbots’ responses is high. New versions tend to perform better than the older ones. Future chatbot advancements may rectify these deficiencies.

## 5. Challenges and Future Directions in Ophthalmology Chatbots

### 5.1. Ethical and Legal Considerations

#### 5.1.1. Privacy and Data Security

Privacy and data security are critical concerns in the context of ophthalmology chatbots, particularly when considering the diverse legal frameworks across different nations. These chatbots gather and process sensitive patient information and personal identifiers, necessitating robust security measures to safeguard these data from unauthorized access, breaches, or misuse.

Ensuring adherence to industry standards and best practices for data encryption and storage is fundamental. Employing encryption techniques, such as secure socket layer (SSL) encryption, can protect the transmission of data. However, developers must also navigate the complexities of varying national laws, which often include additional rules in the field of transmissions, servers, administration, and telecommunication standards. This necessitates a flexible approach to compliance, ensuring that chatbots meet the specific legal requirements of each jurisdiction in which they operate.

Healthcare organizations and developers should establish explicit protocols for data access and sharing, considering different legal landscapes. Transparency in data handling practices is crucial to foster trust among patients, healthcare professionals, and chatbot providers.

To mitigate potential privacy risks and comply with diverse legal standards, privacy-by-design principles should be integrated into the development process. This involves incorporating privacy features from the outset, such as anonymization and data minimization techniques, which are essential in addressing the varied legal requirements across nations (Table 4).

#### 5.1.2. Informed Consent and Confidentiality

Obtaining informed consent and ensuring confidentiality are pivotal ethical considerations when utilizing ophthalmology chatbots. Patients should be fully informed about the purpose, capabilities, and limitations of the chatbot, as well as the type of data it collects and how that data will be used. Informed consent should be sought before engaging patients in chatbot interactions and data collection.

Furthermore, ophthalmology chatbots should provide patients with explicit options to opt in or out of data collection and sharing. Patients should have the ability to withdraw their consent and request the deletion of their data at any time. This empowers patients to exercise control over their personal information, fostering transparency and respecting patient autonomy.

Confidentiality is equally crucial in maintaining patient trust and complying with ethical and legal standards. Ophthalmology chatbots must adhere to stringent confidentiality protocols to ensure that patient data are accessible only to authorized individuals involved in healthcare provision. Measures such as encryption, secure data transmission, and restricted data access help preserve the confidentiality of patient information.

Additionally, chatbots should be programmed to provide appropriate disclaimers and warnings regarding the limitations of their capabilities. Patients should be aware that chatbots do not replace in-person consultations with healthcare professionals and that they should seek medical advice when necessary. This ensures that patients understand the boundaries of chatbot interactions and prompt them to seek appropriate care when needed.

#### 5.1.3. Compliance with Regulatory Standards

Compliance with regulatory standards is essential for ophthalmology chatbots, to ensure patient safety, quality of care, and legal compliance. These chatbots must adhere to relevant regulations and guidelines, such as the Health Insurance Portability and Accountability Act (HIPAA) in the United States and the General Data Protection Regulation (GDPR) in the European Union.

Thorough assessments should be conducted by healthcare organizations and developers of ophthalmology chatbots to ensure compliance with these regulations. This involves reviewing and aligning data handling practices, security measures, and consent procedures with the requirements stipulated in the regulations. Regular audits and assessments can identify any gaps in compliance and facilitate necessary adjustments.

Moreover, collaboration among healthcare organizations, chatbot developers, and regulatory bodies is pivotal in establishing guidelines and standards specific to ophthalmology chatbots. These guidelines should address concerns such as data privacy, informed consent, and the ethical use of chatbots in ophthalmology. By working together, stakeholders can ensure that chatbots meet the necessary ethical and legal standards, while maximizing their potential benefits.

Looking to the future, the development of standardized frameworks and guidelines for ophthalmology chatbots can provide a roadmap for ethical and legal compliance. These frameworks should address the unique challenges and considerations associated with ophthalmology, guaranteeing that chatbots align with the specific needs and requirements of the field. Additionally, ongoing research and evaluation of ophthalmology chatbots can help identify and address emerging ethical and legal issues. Regular monitoring of the evolving regulatory landscape and continuous improvements in chatbot technology can facilitate the development of adaptable and ethically sound solutions.

### 5.2. Integration with Existing Healthcare Systems

#### 5.2.1. Interoperability and Integration Challenges

Ophthalmology chatbots require seamless interoperability and integration with diverse healthcare systems, including electronic medical records, diagnostic devices, and telehealth systems, for efficient usage and effective usage.

One of the primary challenges lies in the diversity of existing healthcare systems, each with its own compatibility level. In addition, diagnosis in the field of ophthalmology frequently requires the integration of multimodality instruments, such as tonometry, perimetry, fundus photography, and optical coherence tomography. Ophthalmology chatbots need to be designed in a way that enables communication and data exchange with various software applications and databases. This necessitates adherence to standardized data formats and protocols that facilitate smooth interoperability.

Ophthalmology chatbots should also be compatible with a variety of devices and operating systems (e.g., desktops, mobile devices). Compatibility across a wide range of devices ensures accessibility and usability for healthcare professionals in clinics, hospitals, and remote sites.

To overcome these challenges, collaboration among healthcare organizations, chatbot developers, and technology providers becomes imperative. The development of standardized application programming interfaces (APIs) and protocols specifically tailored to ophthalmology can expedite the seamless integration of chatbots with existing healthcare systems. The establishment of common standards helps minimize interoperability barriers, thereby enabling efficient data exchange and communication between chatbots and other healthcare tools.

#### 5.2.2. Collaboration with Electronic Health Records

Collaboration with electronic health records (EHR) that contain comprehensive patient information allows chatbots to access and update information in real time. This enhances their capacity to provide personalized and accurate care. However, challenges arise when it comes to EHR integration. Different healthcare organizations may utilize diverse EHR systems, each characterized by a unique data structure and interface. This variability poses a challenge in developing chatbots capable of seamlessly interacting with a wide range of EHR systems.

One potential solution is the development of standardized data exchange formats such as fast healthcare interoperability resources (FHIR), which promote interoperability between EHRs and chatbots. FHIR facilitates the exchange of structured health data, enabling chatbots to retrieve and update patient information from EHR systems in a standardized and consistent manner. Furthermore, collaboration between developers of ophthalmology chatbots and EHR vendors plays a crucial role. Close cooperation can lead to the development of specific interfaces and integration solutions tailored to the field of ophthalmology, thereby ensuring smooth data exchange and seamless connectivity between chatbots and EHR systems.

#### 5.2.3. Seamless Communication with Healthcare Providers

Effective care requires seamless communication between ophthalmology chatbots and healthcare providers. Chatbots should facilitate easy and efficient information exchange, enabling healthcare professionals to review patient data, provide guidance, and make well-informed decisions.

One challenge in achieving seamless communication lies in presenting information in a format that is easily comprehensible and actionable for healthcare providers. Chatbots should present patient data and clinical recommendations concisely and in an organized manner, allowing healthcare providers to quickly grasp the relevant information. The incorporation of NLP capabilities can assist in understanding and presenting complex medical information. Moreover, ophthalmology chatbots should enable bidirectional communication between healthcare providers and the chatbot system. This allows healthcare providers to provide additional context, clarify patient information, and request specific actions from the chatbot. The seamless integration of chatbots with the messaging platforms utilized by healthcare professionals, such as secure messaging applications, can facilitate real-time communication and collaboration.

Ensuring the security and privacy of communications is also of utmost importance. Chatbot systems should employ secure communication channels and encryption techniques to safeguard sensitive patient information during interactions with healthcare providers. Compliance with relevant privacy regulations, such as HIPAA, is essential in upholding patient confidentiality and meeting legal requirements.

To address these challenges, collaboration between chatbot developers and healthcare providers is indispensable. Employing user-centered design methodologies can facilitate the gathering of feedback and insights from healthcare professionals, ensuring that chatbots are designed with their workflow and communication needs in mind. Regular feedback loops and iterative improvements can enhance the usability and effectiveness of chatbot communication with healthcare providers.

### 5.3. Advancements and Future Innovations 

#### 5.3.1. Artificial Intelligence and Machine Learning in Chatbots

**Figure 14:**
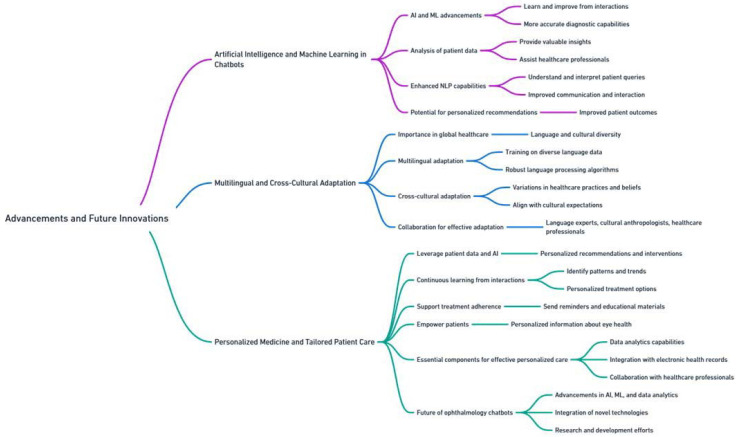
Future studies in the utilization of ophthalmology chatbots.

AI and ML are driving advancements in ophthalmology chatbots, enabling them to learn and improve from interactions with patients and healthcare providers. This leads to more accurate and effective diagnostic capabilities (Figure 14).

Through the analysis of vast amounts of patient data, chatbots can provide valuable insights and assist healthcare professionals in making informed decisions regarding patient care. AI and ML also enhance the NLP capabilities of chatbots. This allows them to understand and interpret patient queries and responses more effectively, facilitating improved communication and interaction. As AI and ML algorithms continue to advance, chatbots have the potential to provide increasingly accurate and personalized recommendations, leading to improved patient outcomes.

#### 5.3.2. Multilingual and Cross-Cultural Adaptation

Multilingual and cross-cultural adaptation is a significant advancement in ophthalmology chatbots, particularly in the context of global healthcare, where language and cultural diversity are prevalent. Chatbots that can effectively communicate and interact with patients from different linguistic and cultural backgrounds improve access to care and enhance patient satisfaction (Table 5).

Developing chatbots capable of multilingual adaptation involves training them on diverse language data and implementing robust language processing algorithms. This enables chatbots to understand and respond to patient inquiries in multiple languages, breaking down language barriers and enabling effective communication.

In addition to language diversity, cross-cultural adaptation is crucial to account for variations in healthcare practices, beliefs, and cultural norms. Chatbots can be programmed to adapt their responses and recommendations based on cultural considerations, ensuring they align with patients’ cultural expectations and preferences. This promotes trust and engagement, ultimately leading to better patient experiences.

Successful multilingual and cross-cultural adaptation requires collaboration with language experts, cultural anthropologists, and healthcare professionals from diverse backgrounds. Such collaboration helps identify specific linguistic and cultural nuances that should be incorporated into chatbot design and development, ensuring cultural sensitivity and effectiveness in different contexts.

#### 5.3.3. Personalized Medicine and Tailored Patient Care

Personalized medicine and tailored patient care hold great promise in the field of ophthalmology chatbots. By utilizing patient data and AI algorithms, chatbots can offer personalized recommendations and interventions tailored to individual patient characteristics, preferences, and medical history.

Through continuous learning from patient interactions, chatbots can gather and analyze data to identify patterns, trends, and personalized treatment options. Chatbots can also support treatment adherence by sending reminders and tailored educational materials, to meet the needs individual of patients. Moreover, chatbots empower patients by providing personalized information about eye health, treatment options, and lifestyle modifications, promoting active participation in their care.

To realize effective personalized medicine and tailored patient care, it is essential to possess robust data analytics capabilities, integrate with electronic health records, and collaborate with healthcare professionals. Developing advanced algorithms that can process and interpret large volumes of patient data, while simultaneously ensuring privacy and security, is of critical importance. Collaborative efforts between chatbot developers and ophthalmologists can refine algorithms and ensure that personalized recommendations align with clinical guidelines and best practices. Looking ahead, advancements in AI, ML, and data analytics will continue to shape the future of ophthalmology chatbots. Integration of novel technologies like computer vision and deep learning holds potential for more accurate and efficient diagnosis of eye conditions. Ongoing research and development efforts are focused on improving chatbots’ ability to handle complex medical scenarios and provide comprehensive and personalized patient care.

In conclusion, advancements and future innovations in ophthalmology chatbots offer exciting opportunities for transforming eye care delivery. Integration of AI and ML can enhance diagnostic capabilities, while multilingual and cross-cultural adaptation can enable effective communication with diverse patient populations. Personalized medicine and tailored patient care promote improved patient outcomes. Continuous research, collaboration, and technological advancements will drive the evolution of ophthalmology chatbots, ultimately benefiting both patients and healthcare providers in the field of ophthalmology.

## Figures and Tables

**Table 1:** Ophthalmology chatbots for patient education and information provision.

Case Scenario	Description	Advantages
**1. Glaucoma Diagnosis**	The chatbot assists patients in comprehending the diagnostic process for glaucoma, elucidating various tests such as tonometry and visual field tests. It imparts knowledge to patients regarding the condition, its symptoms, and available treatment options.	– Enhances patient awareness regarding glaucoma. – Provides precise and consistent information. – Empowers patients to make informed decisions regarding their treatment.
**2. Prevention of Diabetic Retinopathy**	The chatbot educates diabetic patients about the significance of regular eye examinations, early indicators of diabetic retinopathy, and risk factors. It offers recommendations on maintaining optimal blood sugar levels and lifestyle adjustments to minimize the risk.	– Encourages proactive eye care among individuals with diabetes. – Raises awareness regarding the connection between diabetes and ocular health. – Promotes preventive measures to mitigate complications.
**3. Preparation for Cataract Surgery**	The chatbot guides patients through the pre-operative process of cataract surgery, elucidating the procedure, potential intraocular lens options, and post-operative care. It addresses common concerns such as anesthesia, recovery time, and potential risks.	– Reduces anxiety and uncertainty associated with surgery. – Provides comprehensive guidance for pre and post-operative care. – Ensures patients possess realistic expectations and are well-prepared.
**4. Management of Age-related Macular Degeneration (AMD)**	The chatbot furnishes information about AMD, encompassing risk factors, symptoms, and treatment options. It assists patients in understanding the significance of regular monitoring, lifestyle modifications, and available support resources.	– Empowers patients to actively manage their AMD. – Offers resources for emotional support and coping strategies. – Promotes adherence to treatment plans.
**5. Pediatric Eye Conditions**	The chatbot educates parents about prevalent pediatric eye conditions such as amblyopia (lazy eye) and strabismus (crossed eyes). It elucidates the importance of early intervention, available treatment options, and the role of regular eye examinations for children.	– Enhances parental awareness regarding pediatric eye health. – Encourages timely diagnosis and intervention. – Addresses common concerns and dispels misconceptions.

**Table 2:** Example of an ophthalmology discharge summary generated by ChatGPT 3.5.

Prompt	Please write discharge summary for Mr. Somchai Jade, who had a left eye superior retinal detachment involving macular and underwent 25G pars plana vitrectomy with fluid air exchange and endolaser and C3F8 injection, 3 days ago. On the day of discharge, the retina is attached, wound is secure, ac cell is 2+, gas filled 100%, but the intraocular pressure is 25 mm of Hg. He is now initiated on anti-glaucoma medication, Timolol twice a day, in the left eye, instructed to keep face down position and need a follow-up after 5 days at the retina clinic, outpatient clinic with Dr. Ray.
ChatGPT’s response	Patient Name: Mr. Somchai Jade Date of Discharge: [Date] Admitting Physician: [Physician’s Name] Discharging Physician: [Physician’s Name] Diagnosis: Left eye superior retinal detachment involving macular Procedure: Mr. Somchai Jade underwent a 25G pars plana vitrectomy with fluid air exchange and endolaser, followed by C3F8 injection, three days ago. Condition on Discharge: On the day of discharge, the following observations were made:
	Retina: The retina is attached after the surgical procedure. Wound: The wound is secure and healing well. Anterior Chamber: Anterior chamber cell (ac cell) count is 2+. Gas Fill: The eye is filled with 100% C3F8 gas. Intraocular Pressure (IOP): The intraocular pressure is currently elevated at 25 mm of Hg.
	Treatment and Medication: Mr. Jade has been initiated on anti-glaucoma medication, Timolol, to manage the elevated intraocular pressure. He is instructed to administer Timolol twice a day in the left eye, as prescribed. Detailed instructions on the proper administration technique and potential side effects of the medication have been provided to the patient. Additionally, Mr. Jade is instructed to maintain a face-down position as much as possible during his recovery period. This position aids in the proper positioning of the gas bubble in the eye, ensuring optimal healing and attachment of the retina. The patient is advised to follow the specific instructions provided by the surgeon regarding the duration and frequency of the face-down position. Follow-up: Mr. Somchai Jade is advised to follow up with Dr. Ray, a retina specialist, at the retina clinic in the outpatient department after five days. The purpose of the follow-up visit is to assess the progress of the retinal attachment, monitor the intraocular pressure, and evaluate the need for further treatment or adjustment of the medication regimen. Instructions:
	The patient should continue with the prescribed anti-glaucoma medication, Timolol, as instructed. Mr. Jade should maintain a face-down position as directed by the surgeon. Specific instructions regarding the duration and frequency of this position should be followed strictly. It is important to protect the eye from any trauma or injury. The patient should maintain good hygiene and avoid rubbing or touching the eye unnecessarily. In case of any sudden changes in vision, severe pain, or worsening symptoms, Mr. Jade should seek immediate medical attention.
	Summary: Mr. Somchai Jade underwent a successful 25G pars plana vitrectomy with fluid gas exchange and endolaser, along with C3F8 injection, for a left eye superior retinal detachment involving macular. On the day of discharge, the retina was found to be attached, the wound secure, and the anterior chamber showing a moderate cell reaction. The intraocular pressure was elevated, and the patient was initiated on Timolol eye drops for glaucoma management. In addition, Mr. Jade has been instructed to maintain a face-down position during the recovery period to optimize retinal healing. A follow-up appointment has been scheduled after five days with Dr. Ray at the retina clinic to monitor the patient’s progress and adjust the treatment plan if necessary.

Note: This discharge summary is a simulated case created for demonstration purposes only and has not been derived from an actual patient case. It is intended to showcase the capabilities of ChatGPT 3.5 in generating medical content.

**Table 3:** Studies assessing chatbot performance in ophthalmology knowledge assessment.

Study	Mihalache et al. [45]	Raimondi et al. [46]	Bernstein et al. [47]
Study Designs and Population	Cross-sectional study assessing ChatGPT’s performance on ophthalmology board certification practice questions.	Comparative analysis of LLM chatbots on the Fellowship of Royal College of Ophthalmologists (FRCOphth) postgraduate exams.	Cross-sectional study evaluating the quality of ophthalmology advice by ChatGPT compared to ophthalmologists.
Methods	125 text-based multiple-choice questions from OphthoQuestions were used.	Tested on 48 Part 1 and 43 Part 2 multiple choice questions from the FRCOphth curriculum.	200 online forum posts with patient eye care questions and responses were analyzed.
Key Results	– Correct answers: 58 of 125 questions (46%) in January 2023. – Correct answers: 73 of 125 multiple-choice questions (58%) and 42 of 78 stand-alone questions (54%) without multiple-choice options in February 2023.	– Accuracies for chatbots: Part 1 ranged from 55.1–78.9% and Part 2 ranged from 49.6–82.9%. – Bing Chat had the highest scores of 78.9% and 82.9% for Part 1 and Part 2, respectively.	– Expert reviewers identified chatbot vs. human responses with 61% accuracy. – Incorrect information: chatbot 21% vs. human 19%. – Likelihood of harm: chatbot 13% vs. human 15%. – Extent of harm: chatbot 3% vs. human 3%.
Conclusion	ChatGPT answered approximately half of the questions correctly. It may not provide substantial assistance in preparing for board certification currently.	LLM chatbots can achieve high accuracy on ophthalmology specialty exams without specific tuning. They have potential to advance ophthalmic education and care but issues like validation, transparency, biases, and accessibility need addressing.	Chatbot’s ophthalmology advice was not significantly different from ophthalmologists’ advice. LLMs may be capable of providing appropriate responses to patient eye care questions. Further research is needed.

Abbreviations: LLM—large language model; FRCOphth—Fellowship of Royal College of Ophthalmologists.

**Table 4:** Privacy and data security: case scenarios and suggested solutions in ophthalmology chatbots.

Case Scenario: Unauthorized Access to Patient Data	Suggested Solutions
Privacy and data security breach occurs when unauthorized individuals access patient data in the ophthalmology chatbot system.	– Implement strong authentication methods, such as multi-factor authentication.
	– Encrypt patient data both at rest and in transit using strong encryption algorithms.
	– Implement role-based access control mechanisms to restrict access based on job roles.
	– Conduct regular security audits and penetration testing to identify vulnerabilities.
	– Provide comprehensive training to personnel on privacy and data security best practices.
**Case Scenario: Data Breach during Data Transfer**	**Suggested Solutions**
Data breach occurs when patient data are compromised during transmission in ophthalmology chatbots.	– Use secure protocols (e.g., HTTPS, SSL/TLS) for data transmission.
	– Implement data loss prevention mechanisms to monitor and control data transfers.
	– Keep software and systems up to date with the latest security patches and updates.
	– Use secure and well-tested APIs for data exchange with external systems.
	– Encrypt patient data during transmission for an additional layer of protection.
**Case Scenario: Inadequate Data Retention Policies**	**Suggested Solutions**
The ophthalmology chatbot system retains patient data for longer than necessary.	– Implement data minimization practices to collect and store only necessary patient data.
	– Regularly review and delete outdated or unnecessary patient data from the system.
	– Consider anonymizing or de-identifying patient data to protect privacy.
	– Establish clear data retention policies and guidelines for different types of data.
	– Stay updated with privacy laws and regulations and ensure compliance with data retention policies.

**Table 5:** Gaps and Research Opportunities in Ophthalmology Chatbots.

Area of Advancement	Current Gaps	Research Opportunities
AI and Machine Learning in Chatbots.	Limited accuracy in diagnosing eye diseases and conditions; challenges in interpreting ophthalmic data.	Developing advanced AI algorithms for precise diagnosis of eye conditions; enhancing ML capabilities for interpreting complex ophthalmic imaging and data.
Multilingual and Cross-Cultural Adaptation.	Difficulty in addressing language barriers and cultural differences in patient interactions, especially in diverse ophthalmology practices.	Creating chatbots capable of understanding and responding in multiple languages; incorporating cultural sensitivity in patient interactions for global ophthalmology care.
Personalized Medicine and Tailored Patient Care	Lack of personalized treatment recommendations based on individual eye health profiles; limited integration with ophthalmology-specific electronic health records.	Utilizing patient-specific data, to offer personalized eye care recommendations; improving chatbot integration with ophthalmology EHR systems for tailored patient management.

Abbreviations: AI—artificial intelligence; ML—machine learning; EHR—electronic health records.

## Data Availability

Data supporting this study are available in the original publication, reports, and preprints that are cited in the reference citation.
